# Electronic structure, transport, and collective effects in molecular layered systems

**DOI:** 10.3762/bjnano.8.209

**Published:** 2017-10-06

**Authors:** Torsten Hahn, Tim Ludwig, Carsten Timm, Jens Kortus

**Affiliations:** 1 Institute of Theoretical Physics, TU Freiberg, Leipziger Str. 23, D-09599 Freiberg, Germany; 2 Institute of Theoretical Physics, Technische Universität Dresden, 01062 Dresden, Germany

**Keywords:** electron correlation, electronic structure, quantum transport, spin transport

## Abstract

The great potential of organic heterostructures for organic device applications is exemplified by the targeted engineering of the electronic properties of phthalocyanine-based systems. The transport properties of two different phthalocyanine systems, a pure copper phthalocyanine (CoPc) and a flourinated copper phthalocyanine–manganese phthalocyanine (F_16_CoPc/MnPc) heterostructure, are investigated by means of density functional theory (DFT) and the non-equilibrium Green’s function (NEGF) approach. Furthermore, a master-equation-based approach is used to include electronic correlations beyond the mean-field-type approximation of DFT. We describe the essential theoretical tools to obtain the parameters needed for the master equation from DFT results. Finally, an interacting molecular monolayer is considered within a master-equation approach.

## Introduction

Implementing molecular spintronics requires the understanding and the ability to modify and control charge-transport characteristics of organic molecules. Thus a solid understanding of the basic effects that govern the transport characteristics in the desired material is required for the development of further devices. Examples were demonstrated for a wide variety of applications including molecular spin filters [1], single-molecule or thin-film-based field-effect transistors [2–4], as well as potential candidates for memory devices utilizing organometallic complexes of tetracyanoquinodimethane (TCNQ) [5–6]. At interfaces between different organic materials interesting physical phenomena appear, in most cases due to (partial) charge transfer between the materials. One example is the formation of a two-dimensional metallic interface between insulating organic crystals [7–8]. Other effects are metal-insulator transitions or superconductivity which were reported for organic charge-transfer crystals realized by a combination of strongly electron-accepting and strongly electron-donating molecules [9–10]. Recently, a heterostructure of manganese phthalocyanine (MnPc) and structurally similar fluorinated copper phthalocyanine (F_16_CoPc), has demonstrated the occurrence of hybridization [11]. It was proved that a local charge transfer which affects only the transition-metal centers changes the charge state of the transition metal and is directly related to a change of its magnetic moment. Further studies indicated that the Co 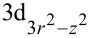 orbital is filled due to the charge transfer at the interface to MnPc. Experiments and theory showed that a bulk material can be formed that maintains the charge and spin transfer between the two molecules [12]. Similar observations were made for organic molecules combined with the strong acceptor molecule F_4_TCNQ. In general all of the fabricated heterostructures revealed new low-energy optical excitations originating from hybrid states. These states are of special importance for the transport characteristics of the hybrid materials. In contrast to other organic molecules, the hybrid dimer states close to the Fermi level in the the picene/F_4_TCNQ compound excite a very asymmetric *I*–*V* curve with a pronounced diode-like forward/reverse current behavior. Additinally the effect of an applied gate voltage is greatly enhanced [13].

The electronic structure of free molecules or molecular assemblies will be substantially modified if the molecular material comes in contact with metal substrates. The formation of hybrid states at the metal-organic interface due to the different chemical potentials of the materials induces a wide range of effects and strongly depends on the microscopic details of the interface. The question arises of how the substrate interactions change the electronic structure of the molecular material and whether favorable properties for envisaged applications can be realized. Another important aspect for transport and potential applications are electronic interactions and correlations, which can be very strong in the confined molecular orbitals. Approaches beyond mean-field-type approximations are required for the treatment of correlation effects such as Coulomb blockade and the Kondo effect [14]. Such interactions not only occur within a single molecule but also between neighboring molecules in a film [15], where they can lead to ordering phenomena.

Our paper is organised as follows. First we will present the methodical background and results of our theoretical investigations on different phthalocyanine heterostructures by using the DFT-NEGF approach. In the second part we present our approach to combine DFT calculations and the master equation approach to quantum transport. Finally we present results of this new approach to describe tunnelling effects in monolayers.

## DFT-NEGF transport theory

The ground-state electronic structure of the molecules was investigated using the all-electron DFT NRLMOL program package, which achieves a high level of numerical accuracy (see [16–17] and references therein). For the exchange correlation, GGA/PBE [18] was used and in all calculations dispersion correction utilizing the DFT-D2 method [19] was included. The geometry of the molecules was optimized using a gradient approach, the relaxation was terminated once all atomic were below 0.05 eV/Å. We applied the NEGF method for the self-consistent calculation of the electronic transport properties as implemented in the GPAW code [20–21] to investigate the *I*–*V* characteristics of our model devices. For the transport calculations, the electronic structure is obtained by DFT calculations using the common approach of constructing a model device for which the molecule of interest together with additional electrode atoms (scattering region, see below Figure 2e) are sandwiched between two semi-infinite metallic electrodes. We use at least three additional Au(111) layers at each side of the molecule to construct the scattering region, followed by a further geometry-optimization step, where the topmost two gold layers together with the attached molecules are allowed to relax. For the scattering region as well as for the leads, a localized double-ζ polarized basis set was used. The whole system can be subject to an external bias and/or gate voltage. The electronic structure of the scattering region and therefore the *I*–*V* curves are calculated self-consistently in the presence of such external fields.

The key facts of the DFT-NEGF method where already given in [13] and a detailed discussion of the method can be found in the cited literature and the references therein [22–23].

### Ground state molecular properties

Important effects arise from interactions between the organic molecules and metallic contacts. These interactions may substantially alter the electronic structure of the organic material and needs to be carefully investigated [24]. In the following, we present DFT results for model systems were two phthalocyanine systems are in contact with Au(111) and Ni(111) surfaces.

We have investigated a F_16_CoPc/MnPc heterostructure, which exhibits ground-state charge and spin transfer. We compare the results to a CoPc/CoPc reference structure, which does not show spin and charge transfer effects in the ground state. For both organic materials, we assume β-stacking [25].

The selected Au surface is known to form metal–organic contacts with medium interactions [26]. On the other hand, pure Ni surfaces are known to have a very high reactivity that sometimes lead to decomposition of the deposited organic material [27–28]. The reactivity of the Ni contact can be reduced by inserting a single layer of graphene between the organic molecule and the metal surface.

The model systems used here were built by first relaxing the F_16_CoPc/MnPc and CoPc/CoPc molecular stacks on top of five-layer metal slabs. In a second step, the model device was built by adding a second metal slab on top of the organic material, with subsequent relaxation. The distance between the second contact and the organic material was systematically varied and the structure with the lowest total energy was used for the transport calculations.

**Figure 1:**
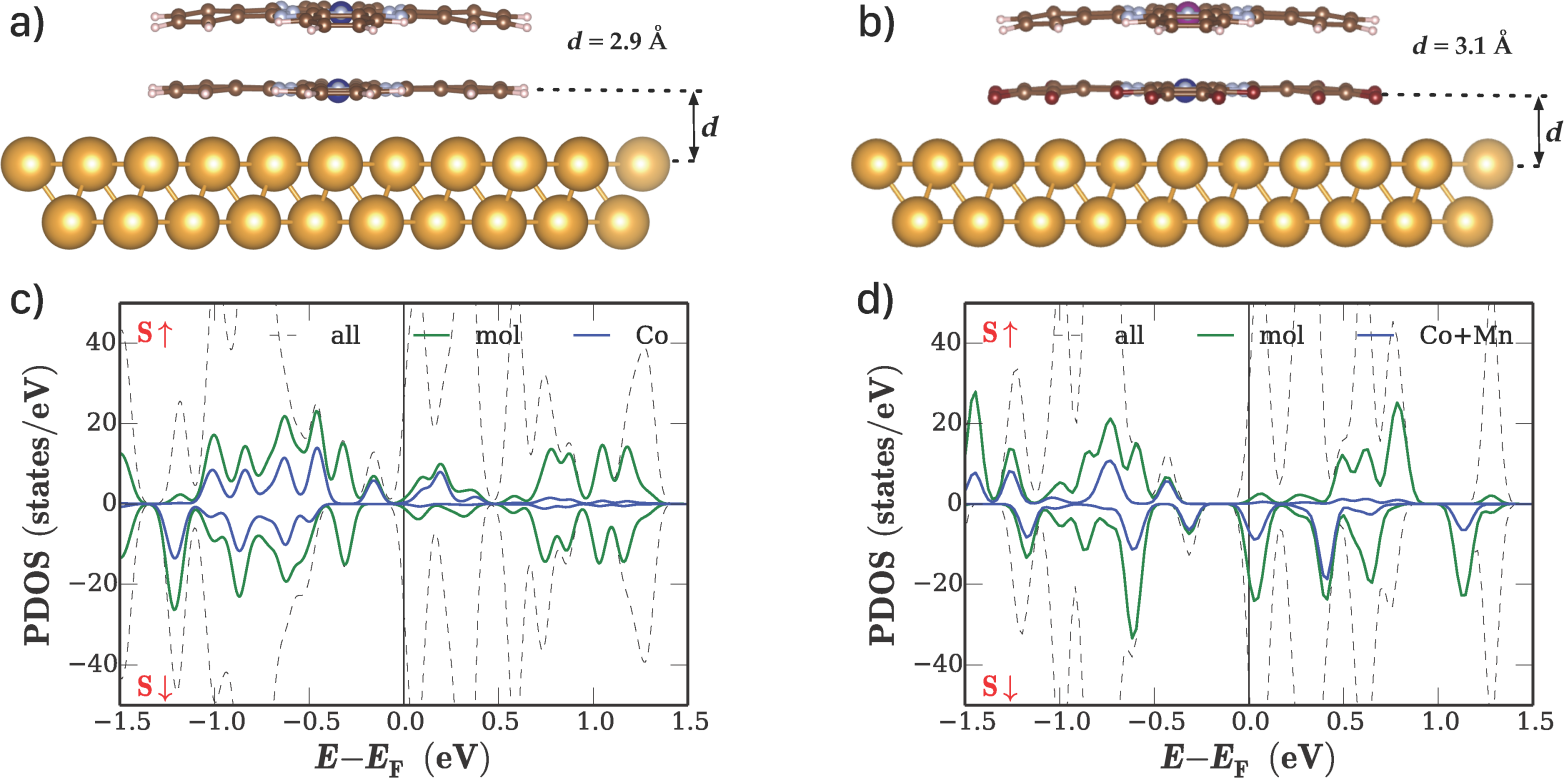
Results of DFT calculations for phthalocyanine stacks on fcc-Au(111) surfaces: relaxed geometries of a) the CoPc/CoPc stack and b) the F_16_CoPc/MnPc stack. c), d) Density of states (DOS) of the molecule-Au(111) interfaces as obtained from the calculations. The overall DOS as well as the projections onto the molecule and metal centers are shown.

In Figure 1, we show the results for the two organic systems between Au(111) surfaces. The electronic properties of both systems are altered due to the interaction with the gold surface. While in the contact-free CoPc/CoPc stack, the cobalt atoms couple antiferromagnetically, yielding an *S* = 0 system, the interaction with the gold surface reduces the Co moment due to a charge transfer from the metal surface. Qualitatively, the same effect is observed for the F_16_CoPc/MnPc stack. Again, charge is transferred from the Au surface to the Co atom, in agreement with experimental results [29]. Figure 1c,d shows the respective plots of the density of states as obtained from the DFT calculations. While the electronic structure of CoPc and F_16_CoPc is qualitative similar after surface contact, the manganese center in the F_16_CoPc/MnPc yields a larger local magnetic moment and more strongly occupied metal 3d states close to the Fermi level. Both structures show some asymmetry between the spin-up and spin-down DOS.

## Results and Discussion

### Transport through phthalocyanine heterostructures

**Figure 2:**
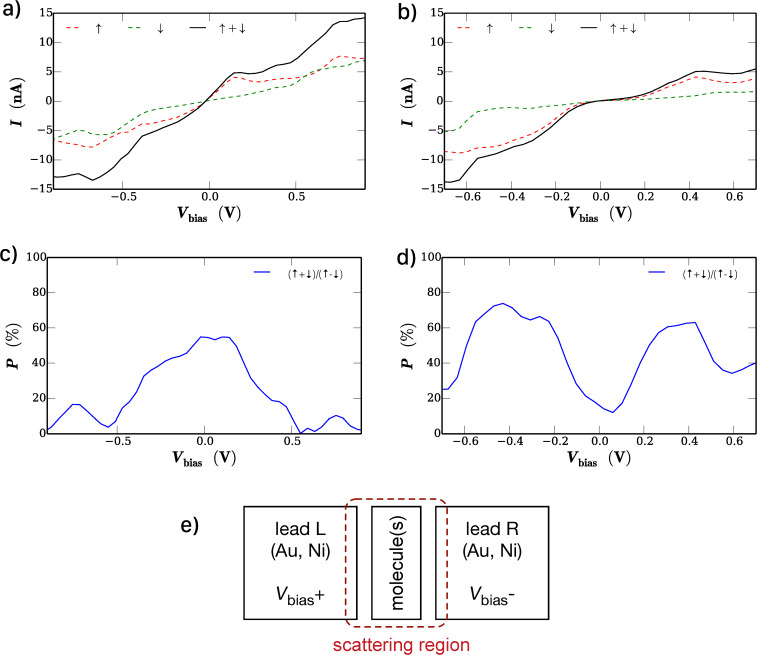
*I*–*V* curves calculated within the DFT-NEGF method for the sandwich structure a) CoPc/CoPc and b) F_16_CoPc/MnPc. c), d) Spin polarization of the current as a function of the bias voltage. e) Schematic drawing of the used device configuration for the DFT-NEGF transport calculations.

The ground state calculation results are reflected in the corresponding *I*–*V* curves shown in Figure 2a,b together with plots of the spin polarization of the current as a function of the bias voltage in Figure 2c,d. As expected, the resulting *I*–*V* curves show pronounced non-linear behavior in both cases and one can identify features in both curves that reflect distinct electronic states of the material. A second important result is the fact that the spin polarization of the current depends strongly on the applied bias voltage. While for the CoPc/CoPc system the spin polarization vanishes with increasing bias voltage, the F_16_CoPc/MnPc stack shows maxima of the spin polarization at approximately *V*_bias_ = ±0.5 V of over 60% and the polarization does not vanish for larger bias voltages.

**Figure 3:**
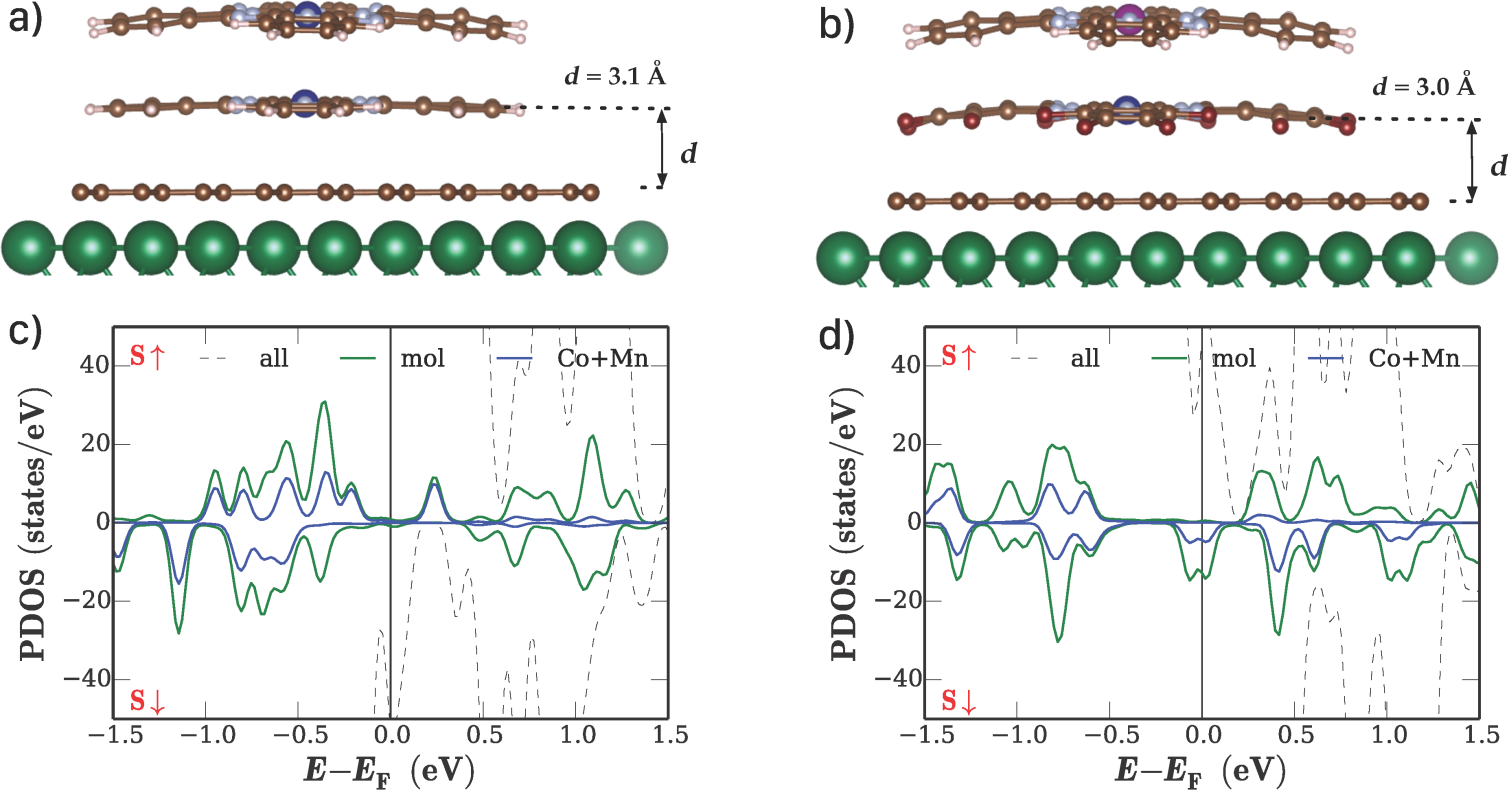
Results of DFT calculations for phthalocyanine stacks on fcc-Ni(111) surfaces: relaxed geometries of a) the CoPc/CoPc stack and b) the F_16_CoPc/MnPc stack. c), d) DOS of the molecule-Au(111) interfaces as obtained from the calculations. The overall DOS as well as the projections onto the molecule and metal centers are shown.

The same methodology is applied to the second model system, where the two different molecular stacks are in contact with magnetic Ni(111) leads. The quantity of interest for possible applications is the tunnel magnetoresistance (TMR), which can be obtained directly from *I*–*V* calculations with parallel and antiparallel magnetization of the Ni leads. The very strong interaction with the ignoble Ni surface leads, however, to a complete loss of the molecular properties of the organic material. For this reason, we introduce a single layer of graphene between the nickel surfaces and the molecular material on both sides of the device. Based on this layout, which is shown in Figure 3a,b, it was possible to obtain device structures for which the geometry of the Pc/Pc stacks was preserved during relaxation. Contrary to the direct deposition on a gold surface, the additional graphene layer effectively decouples the molecular stacks from the reactive Ni surface and preserves the electronic structure of the molecular material. The DOS for both systems is shown in Figure 3c,d.

**Figure 4:**
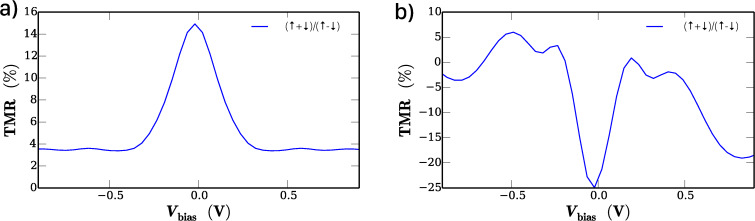
Calculated TMR for the sandwich structure a) CoPc/CoPc and b) F_16_CoPc/MnPc on Ni(111). The TMR as a function of the bias voltage is obtained from the spin-polarized current within the DFT-NEGF method.

The corresponding TMR is shown in Figure 4a,b. The DFT-NEGF methodology produces qualitative different results for the two material systems. Apart from increased values at very low bias voltages, the CoPc/CoPc stack exhibits a rather constant TMR of approximately 4%. On the other hand, the F_16_CoPc/MnPc system shows significantly higher TMR values than the CoPc/CoPc system. Another interesting feature of the F_16_CoPc/MnPc stack is the fact that the TMR changes sign depending on the applied bias voltage, which demonstrates the effect of the molecular properties on the observed current and ultimately on the TMR effect. It was already validated experimentally in [30] that the tunneling through single CoPc molecules on ferromagnetic Fe thin film exhibits pronounced spin dependence.

These investigations suggest the possibility of versatile applications in spintronic devices. The calculations on model systems with gold contacts show that it is possible to obtain spin-polarized currents from both phthalocyanine-based devices. However, the F_16_CoPc/MnPc heterostructure yields a stronger spin polarization of the current, which is predicted not to vanish for high bias voltages. The investigated prototypical TMR device yields a qualitatively consistent picture. The predicted TMR for the F_16_CoPc/MnPc heterostructure is by a factor of 2–3 larger than the TMR of the pure CoPc device, depending on the applied bias voltage. Our results make the F_16_CoPc/MnPc material system a more promising candidate for applications. In principle the experiments presented in [31] did show that application specific design of transport properties is possible by variation of the stack size of CoPc molecules. The use of different types of phthalocyanines as suggested in this work seems to open a new path to design transport properties.

The DFT-NEGF as a standard approach for the investigation of transport properties of model device structures gives reasonable information on whether a specific materials combination is suitable for applications. However, one has to keep in mind that the electronic structure used as input is derived from ground-state DFT results and thus has the limitations inherent to the DFT method. To provide a more comprehensive picture, especially in situations where the electronic correlations are strong, it is necessary to apply techniques that permit a treatment of molecular interactions beyond the mean-field-like DFT approach.

### DFT combined with the master equation

An improved treatment of electronic correlation is relevant especially for weakly hybridized molecular systems since the electrons are confined to relatively small molecular orbitals so that electron–electron interactions dominate. DFT typically gives reasonable results for the spatial structure of orbitals, whereas energy levels are not always well reproduced. Even if the energies are reasonable, the magnitude of the tunneling currents through nanoscale devices are often strongly overestimated [32–35]. The origins of these problems are threefold: First, tunneling under a finite bias is a non-equilibrium situation that is not well described by standard DFT, which is a method for the ground state. In principle, excited states and time-dependent effects can be treated using time-dependent density functional theory and time-dependent current density functional theory [36–37]. However, this is complicated by the lack of good approximate exchange-correlation functionals for transport calculations [38] and by the high computational cost. Second, standard functionals for DFT do not describe strongly correlated systems particularly well. Third, NEGFs can describe tunneling (hybridization) exactly but naturally lead to perturbative approximations for interactions.

The master-equation (ME) approach focuses on the many-body state of the molecular system and traces out the degrees of freedom of the electrodes, e.g., the top and bottom Au or Ni electrodes discussed in the previous sections or the tip and the substrate in an STM setup. We are here interested in the latter situation. The ME is an equation of motion for the reduced density operator ρ_mol_ of the molecule [14,39–53]. The ME approach is complementary to NEGFs in that it allows to treat the interactions within the molecule exactly but lends itself to approximate expansions in the tunneling between the molecule and the leads. The method is thus powerful for strong interactions but weak hybridization between the molecules and the electrodes (STM tip and substrate).

The ME approach requires the formal separation of the system into the molecule and the electrodes, where the connection between them is expressed by a bilinear tunneling Hamiltonian 
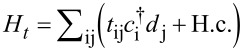
. Here, *t_ij_* are tunneling amplitudes and 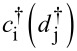 are electronic creation operators for the molecule (the electrodes). The derivation of tunneling amplitudes *t_ij_* from a fundamental interacting Hamiltonian has been studied intensively [54–59] but is still not completely solved [60]. For STM, the tunneling amplitudes describing tunneling between the tip and the molecule or the substrate depend on the tip position.

It is highly desirable to obtain realistic, system-specific tunneling amplitudes based on DFT. While the combination of DFT with NEGFs is integrated in existing packages, not much work has been done for DFT combined with the ME. In the following, we outline the main steps needed for such an approach and illustrate the feasibility by showing results for CoPc on graphene. The Hamiltonian reads *H* = *H*_leads_ + *H*_mol_ + *H_t_*, where

[1]
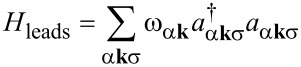


describes the tip (α = *T*) and the substrate (α = *S*). Both are modeled as non-interacting electron gases with DOS *D*_ασ_(ξ) and chemical potentials μ_α_. 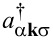 creates an electron in lead α with wave vector **k**, spin σ, and energy ω_α_**_k_** (taking 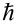 = 1). The molecular part is

[2]
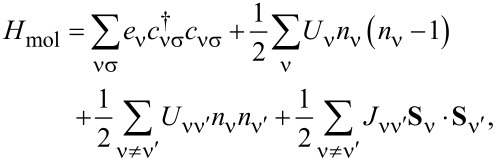


where 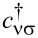 creates an electron in the molecular orbital ν with spin **σ** and single-particle energy *e*_ν_, 
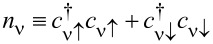
 is the corresponding number operator, and 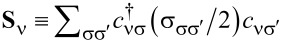 is its spin operator in terms of the vector of Pauli matrices, **σ**. *U*_ν_ and *U*_νν'_ = *U*_ν'ν_ describe the intraorbital and interorbital Coulomb interactions, respectively, and *J*_νν'_ = *J*_ν'ν_ is the Hund-rule coupling. The orbital energies *e*_ν_ are shifted by the electric potential, which is controlled by the bias voltage *V* = (μ*_T_* − μ*_S_*)/*e*.

The eigenenergies and eigenstates of *H*_mol_ satisfy 
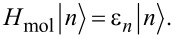
 Only the differences between molecule and electrode energies enter the final results and it is useful to keep the molecular energies unchanged and instead shift the chemical potentials. A simple estimate is given by Datta et al. [23], who model tip and substrate as capacitor plates. For fixed molecular energies the chemical potentials are then μ*_T_* = η*eV* and μ*_S_* = (η − 1)*eV*, where η ≡ *z*_mol_/*z*_tip_. Here, *z*_mol_ is the distance to the molecule and *z*_tip_ the distance to the tip, both measured from the substrate. Thus η can in principle be varied in the range 0 *<* η *<* 1. Better approximations taking account of the actual geometry are of course possible.

Finally, the tunneling between the molecule, the tip, and the substrate is described by

[3]
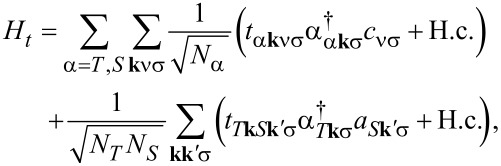


where the first term corresponds to tunneling between the molecule and lead α, while the second corresponds to direct tunneling between tip and substrate. The numbers *N*_α_ of sites in lead α drop out of the physical results.

For calculating the stationary current under an arbitrary tip–substrate bias voltage, we employ the sequential-tunneling approximation, i.e., we expand the ME up to the first non-vanishing order in the tunneling amplitudes. The derivation is standard, see, e.g., [45,47–53]. It starts from the exact von Neumann equation for the full density operator of the tip–molecule–substrate system. Taking the trace over the tip and substrate degrees of freedom, one obtains a ME for the reduced density operator ρ_mol_. The ME is then expanded up to second order in *t*_α_**_k_**_νσ_. For the stationary state, off-diagonal components of ρ_mol_ in the eigenbasis of *H*_mol_ (i.e., coherences) vanish if the system is non-magnetic or all magnetic axes (applied magnetic field, magnetization, easy anisotropy axis) are parallel. Then one obtains rate equations for the diagonal components, i.e., for the probabilities of molecular states,

[4]
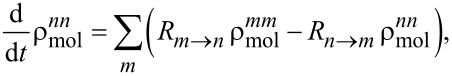


where *m* and *n* label molecular eigenstates and

[5]
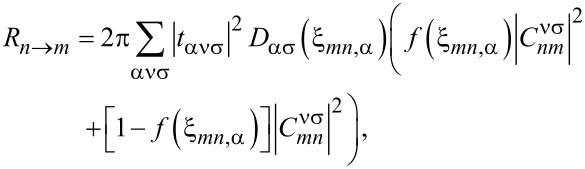


with ξ*_mn,α_* ≡ ε*_n_* − ε*_m_* − μ_α_ are transition rates for sequential tunneling. We have assumed the tunneling amplitudes to be independent of the wave vector **k**. The matrix elements 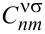 are defined as 
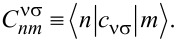
 Finally, the current is

[6]
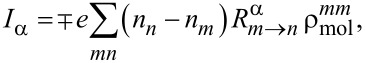


where the upper (lower) sign pertains to α = *T* (*S*), *n_n_* denotes the occupation number in the eigenstate 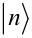, and the rates 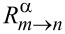 contain only terms involving lead α.

We now turn to the determination of the model parameters from DFT. At least two different charge states must contribute to obtain sequential tunneling but more charge states can be relevant, in particular for large bias voltages. Furthermore, for any charge state, certain orbitals will contribute to sequential tunneling. Their relative energies for the same charge is usually well described by DFT. Energy differences between states with *N* and *N* − 1 electrons are best obtained from the ionization energies of the *N*-electron systems. The DOS *D*_ασ_(ξ) of the tip and the substrate are standard quantities obtained from band-structure calculations.

The calculation of the tunneling amplitudes *t*_ανσ_ is our main concern. We start by considering the molecule–substrate interface. The approach uses DFT to calculate the KS orbitals and eigenvalues and the KS potential of the free substrate, of the free molecule, and of both combined. Similarly to [55,61], we write the Hamiltonian as

[7]
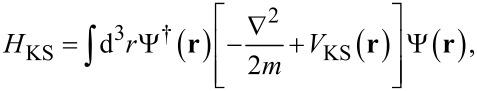


where *V*_KS_ is the KS potential for molecule and substrate combined. We now split the field operator Ψ into two parts according to

[8]
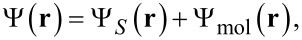


[9]
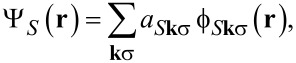


[10]
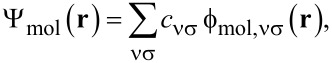


where the 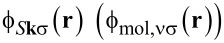 are KS orbitals from the calculation for the substrate (molecule) alone. Each set by itself forms a complete basis of the space of single-particle wave functions. Taken together, they are thus overcomplete so that the decomposition in Equation 8 is not unique. To cure this problem, we only include a (typically small) number of relevant molecular orbitals in Ψ_mol_ and throw out the same number or more of high-energy orbitals from Ψ*_S_*. Which ones these are is irrelevant for the low-energy physics. The remaining wave functions are linearly independent. However, the KS orbitals for the molecule and those for the substrate are not orthogonal. This would make the tunneling amplitudes ill-defined, as we shall see, and we therefore orthonormalize the states. Since our purpose is to identify the orbitals as molecule and substrate states, we demand that the orthonormalized states deviate minimally from the (input) states of the molecule and substrate alone. This is achieved by Löwdin orthonormalization [62–63]. The resulting orbitals are denoted by 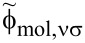 and 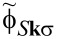 and the corresponding fermion operators by 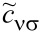 and 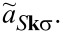


The KS Hamiltonian (Equation 7) is not diagonal in the new basis. Generally, there are off-diagonal components within the sector of molecular states, within the sector of substrate states, and between the two. For the molecular sector, the off-diagonal matrix elements 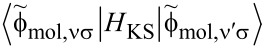 describe the mixing of molecular states due to the presence of the substrate. The coupling to the substrate also leads to a change of the diagonal matrix elements. In principle, all these matrix elements can be absorbed into the model Hamiltonian *H*_mol_. In the substrate sector, the off-diagonal matrix elements 
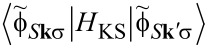
 affect the local DOS at the surface. These effects on the molecule and the substrate lead to higher-order corrections on top of the sequential-tunneling approximation and are neglected to leading order.

The tunneling amplitudes between molecule and substrate are given by 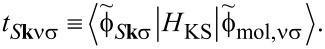 The additional approximation of **k**-independent tunneling amplitudes in Equation 5 requires us to average over **k** or, if the dependence is seen to be weak, choose a representative substrate state.

The orthonormalization of states is crucial: If we had worked with non-orthonormalized wave functions, adding a supposedly irrelevant constant *C* to the Hamiltonian *H*_KS_ in Equation 7 would change *t_S_***_k_**_νσ_ by 
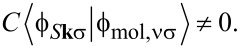
 Then the rates (Equation 5) and, consequently, all observables would depend on *C*. This problem already appears in the seminal paper of Slater and Koster [64]. Using orthonormalized states avoids the ambiguity.

For the tunneling amplitudes between the molecule and the tip, *t_T_***_k_**_νσ_, and between the substrate and the tip, *t_T_***_k_***_S_***_k_**_'σ_, one can use an analogous procedure, with one important modification. It is unfeasible to perform a DFT calculation for every relevant tip position for the full tip–molecule–substrate system. Instead, we take the sum of the KS potentials obtained separately for the substrate, the molecule, and the tip (translated to any tip position of interest) as an approximation for the full KS potential 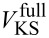. This neglects the interaction of the molecule with substrate and tip for the purpose of calculating the tip–molecule tunneling amplitudes and is valid for weak hybridization. The tip–molecule tunneling amplitudes are finally calculated as

[11]
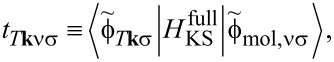


where

[12]
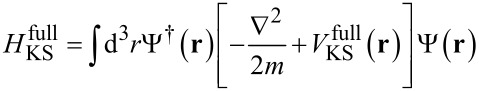


and 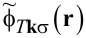 is a properly orthonormalized tip wave function. The calculation of the tip–substrate amplitudes is analogous to the case of the tip–molecule amplitudes, with the molecular wave functions 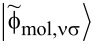 replaced by the substrate wave functions 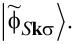


**Figure 5:**
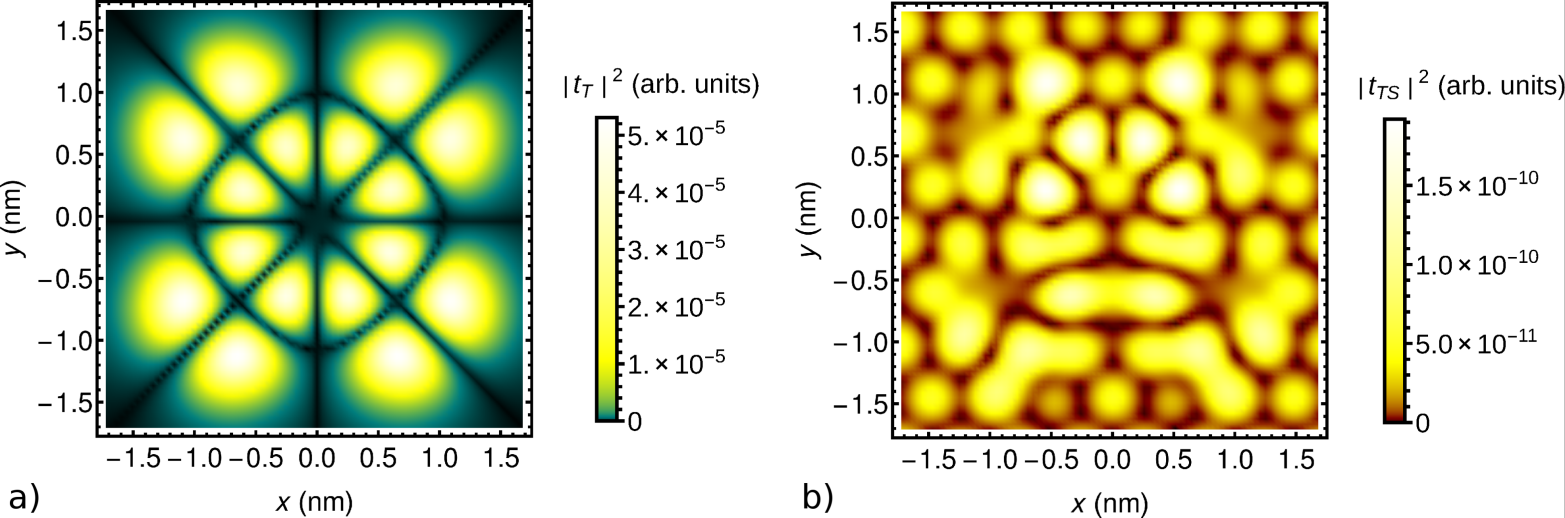
Absolute value squared of the tunneling amplitude a) between the STM tip and a CoPc HOMO on a graphene substrate and b) between the tip and a representative low-energy Bloch-type substrate state, as functions of the lateral position (*x*,*y*) for fixed height *z* = 0.64 nm.

For illustration, we show in Figure 5a the absolute value squared |*t_T_*|^2^ of the tunneling amplitude between the tip and the highest occupied molecular orbital (HOMO), in this case of CoPc, as a function of the lateral position (*x*,*y*) for fixed height *z* = 0.64 nm. The tip was approximated by a single hydrogen 1s orbital for simplicity. The substrate was taken to be a graphene monolayer for simplicity, intended as a decoupling layer as discussed in section ’DFT-NEGF transport theory’. The symmetry of the HOMO is clearly visible and is not noticeably reduced by the hybridization with the substrate. Figure 5b shows the absolute value squared |*t_TS_*|^2^ of the direct tunneling amplitude between the tip and a representative low-energy substrate state, specifically the Bloch state at the *K* point localized on one of the two sublattices, modified by the Löwdin orthonormalization with respect to the CoPc HOMO and the tip. The amplitude is enhanced where large weights of the substrate and CoPc orbitals coincide. The enhancement signifies coherent tunneling from the tip through the molecule to the substrate. Note, however, that the tip–substrate amplitude *t_TS_* is small compared to the tip–molecule amplitude *t_T_* for the present height *z*.

### Tunneling through monolayers

Many molecules form highly ordered self-assembled monolayers on appropriate substrates [65–70]. Sandwich structures of monolayers contacted by conducting materials at the top and bottom are of interest for applications and also from a fundamental point of view since non-local interactions between molecules are relevant. The combination of interactions with a bias voltage perpendicular to the monolayer can lead to interesting non-equilibrium properties. The fabrication of the top contact has proved to be difficult since the technique must be sufficiently gentle not to damage the molecular layer. One successful technique involves rolled-up nanolayers [71–78].

An advanced theoretical description extending the mean-field-type description within the DFT-NEGF approach requires a method that can deal with strong interactions in systems far from equilibrium, and the prime candidate is again the ME approach discussed in Section ’DFT combined with the master equation’, ideally using parameters from DFT calculations. The non-local interaction adds another level of complication [15,79]. In the sequential-tunneling and diagonal approximation described above, this interaction can be treated essentially exactly using Monte Carlo simulations [15]. The main idea is to use the sequential-tunneling rates, which are analogous to Equation 5 and uniform throughout the monolayer, to determine the probabilities of local Monte Carlo updates. Importantly, these rates depend on the total occupation of the neighboring sites through the nearest-neighbor Coulomb interaction. Note that the rates do not satisfy detailed balance for nonzero bias voltages.

A simple model system consisting of a square lattice with a single spinful orbital per site and with very strong intraorbital and arbitrary nearest-neighbor Coulomb interactions has recently been studied by two of us [15]. There, the molecules have been assumed to be symmetric, which would for example be appropriate for a CoPc layer. In the present work, we consider a minimal model for a layer of dimers such as F_16_CoPc/MnPc [11–12] sandwiched between electrodes. F_16_CoPc/MnPc has a twofold spin degenerate HOMO so that a model with a single orbital per site with interactions should be reasonable. The main difference from the previously studied case [15] is the asymmetry of the molecule. The asymmetry can be modeled by assuming different tunneling probabilities between the molecular orbital and the two electrodes. In the following, we analyze how such an asymmetry affects observables and compare to the symmetric case. For details of the theory we refer to [15].

The main parameters of the model are the on-site energy *E_d_*, the nearest-neighbor Coulomb repulsion *U*_1_, and the bias voltage *V*. The on-site Coulomb repulsion *U*_0_ is set to infinity, excluding double occupation. Results are plotted as functions of ratios *E_d_*/*U*_1_ and *eV*/*U*_1_. The ratio Γ_top_/Γ_bottom_ of the tunneling rates Γ_α_ 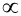 |*t*_α_|^2^ is taken to equal 0.5. We here restrict ourselves to the limit of zero temperature. In this limit, the transition rates are step functions of the molecular energy level *E_d_* and of the bias voltage *V*. Consequently, all observables are also step functions.

Regions that contain a piece of the *V* = 0 axis or touch that axis at their boundary have rates that are the same as for an equilibrium model in the limit of *T* → 0. The stationary state is thus the equilibrium state for *T* → 0, i.e., the ground state. Since the model is of Ising type, with the modification of the two-fold (spin) degeneracy of the occupied single-site states, this ground state is known to be the completely occupied state for *E_d_*/*U*_1_ *<* −4, a state with checkerboard charge order for −4 *< E_d_*/*U*_1_ *<* 0, and the completely empty state for *E_d_*/*U*_1_ *>* 0. The other simple limiting case pertains to sufficiently large bias voltage |*V*|. In this limit, all sequential-tunneling rates are nonzero and are independent of the occupation of the neighboring sites. Thus the layer decouples into independent sites. Moreover, forward and backward rates are always equal, *R_n_*_→_*_m_* = *R_m_*_→_*_n_*, so that the system is equivalent to a model at infinite temperature. For the other regions, we have performed Monte Carlo simulations as in [15].

**Figure 6:**
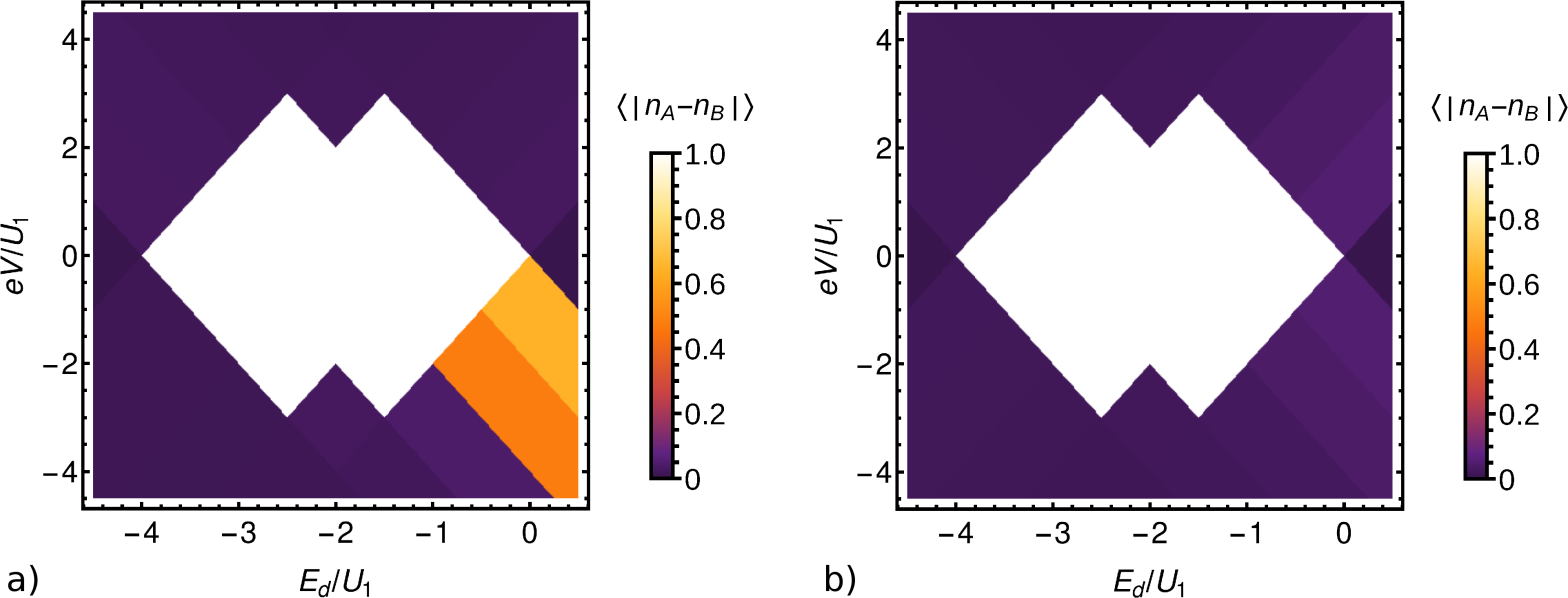
Average imbalance 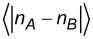 between the occupations *n_A_* and *n_B_* of the two checkerboard sublattices for a) asymmetric tunneling, Γ_top_/Γ_bottom_ = 0.5, and b) symmetric tunneling, Γ_top_/Γ_bottom_ = 1 [15], both for a degeneracy of 2 of occupied single-site states.

**Figure 7:**
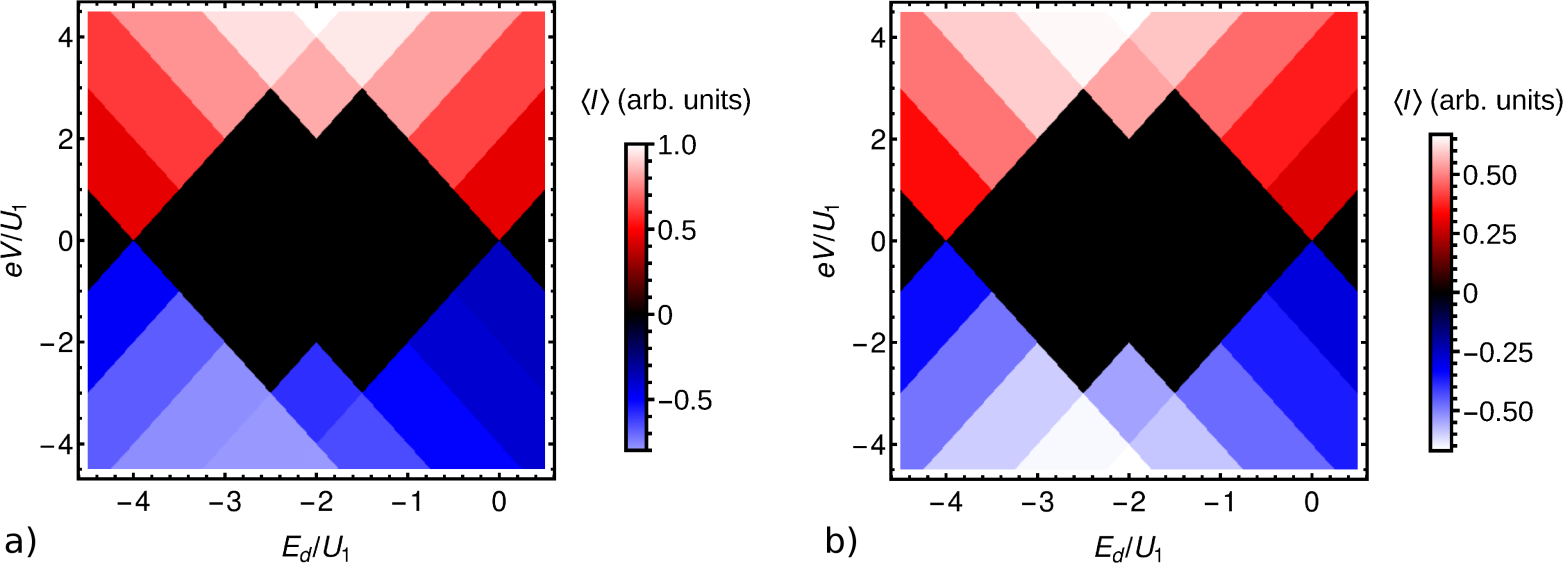
Average current 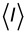 per site for a) asymmetric tunneling, Γ_top_/Γ_bottom_ = 0.5, and b) symmetric tunneling, Γ_top_/Γ_bottom_ = 1 [15], both for a degeneracy of 2 of occupied single-site states.

Figure 6a shows the average imbalance 
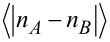
 between the occupations *n_A_* and *n_B_* of the two checkerboard sublattices, for the case of Γ_top_/Γ_bottom_ = 0.5. For comparison, we show the corresponding results for symmetric contacts, Γ_top_/Γ_bottom_ = 1, in Figure 6b [15]. Figure 7 shows the average current per site for both cases. Evidently, there is a phase with checkerboard charge order and vanishing current for both values of the asymmetry. It extends the equilibrium checkerboard ordered phase to nonzero bias voltages *V*. We next note that Γ_top_ ≠ Γ_bottom_ breaks the symmetry between positive and negative bias. The current reaches a larger value for positive bias, the device thus acts as a (rather poor) rectifier. This is expected. Much more interestingly, we find two regions, in the lower right quadrant of Figure 6a, where checkerboard order coexists with a nonzero current. Such a checkerboard conducting phase was predicted in [15]. However, for the symmetric contacts considered there, it only occurs for degeneracies of the occupied sites of at least 4. Such a large degeneracy is hard to realize. The new results show that for a very moderate asymmetry of the device, the spin degeneracy of 2 is already sufficient to stabilize this interesting phase. In this phase, tunneling takes place only through one sublattice, which has an average occupation between 0 and 1, while the other sublattice is empty. According to Figure 6a, it occurs for negative bias voltages, which correspond to electrons tunneling out of the bottom electrode into the molecules. This is the junction with the larger tunneling rate Γ_bottom_. Thus the asymmetry favors in-tunneling from the bottom electrode. Since increasing the degeneracy of the occupied sites also favors the occupied state and this can stabilize the checkerboard conducting phase [15], it is plausible that the asymmetric tunneling has the same effect.

## Conclusion

In this contribution, we have discussed and illustrated approaches to transport calculations for molecular systems sandwiched between conducting electrodes. In the first part, we have reported on the transport properties of two different phthalocyanine structures. Our studies using the standard DFT-NEGF approach show that both structures exhibit transport properties that may be useful for device applications. A reasonable spin polarization of the current through model devices with non-magnetic Au(111) leads is predicted. For F_16_CoPc/MnPc heterostructure, this polarization is more robust at higher bias voltages, which qualifies this hybrid material as the better candidate for a possible spin-filter application. Devices with magnetic Ni(111) contacts yield TMR values of 4% for the pure CoPc system and up to 18% for the F_16_CoPc/MnPc heterostructure at bias voltages relevant for applications. In the second part, we point out that the DFT-NEGF approach becomes questionable if electronic correlations in the molecule are strong, and introduce an alternative approach based on combining DFT with the ME. We discuss how a model suitable for ME calculations could be constructed on the basis of DFT calculations and a first proof-of-concept implementation of coupling DFT and ME is presented. Unlike for the well established NEGF, a lot of work remains to be done, however this could lead to a new way to investigate transport in strongly correlated materials. Finally, we show how strong Coulomb interactions between different molecules in a monolayer sandwiched between electrodes can be treated within a ME approach. This method is applied to asymmetric molecular systems such as F_16_CoPc/MnPc. Besides the expected current rectification, it is found that the asymmetry can lead to a non-equilibrium conducting state with checkerboard charge order.
